# Phytochemical and pharmacological progress on the genus *Syringa*

**DOI:** 10.1186/s13065-015-0079-2

**Published:** 2015-01-27

**Authors:** Guozhu Su, Yuan Cao, Chun Li, Xuelong Yu, Xiaoli Gao, Pengfei Tu, Xingyun Chai

**Affiliations:** Modern Research Center for Traditional Chinese Medicine, Beijing University of Chinese Medicine, 11 North 3rd Ring Road, Chaoyang District Beijing, 100029 P. R. China; School of Chinese Materia Medica, Beijing University of Chinese Medicine, 6 Wangjing Southern Middle Ring Road, Chaoyang District Beijing, 100102 P. R. China

**Keywords:** *Syringa*, Oleaceae, Iridoid, Lignan, Phenylethanoid, Bioactivities, Review

## Abstract

Genus *Syringa*, belonging to the Oleaceae family, consists of more than 40 plant species worldwide, of which 22 species, including 18 endemic species, are found in China. Most *Syringa* plants are used in making ornaments and traditional medicines, whereas some are employed for construction or economic use. Previous studies have shown that extracts of *Syringa* plants mainly contain iridoids, lignans, and phenylethanoids that have antitumor, antihypertensive, anti-oxidant, and anti-inflammatory activities. This study reviews phytochemical and pharmacological progress on *Syringa* in the recent 20 years and discusses the future research prospects to provide a reference in further promotion and application of the genus.

**Graphical Abstract:**
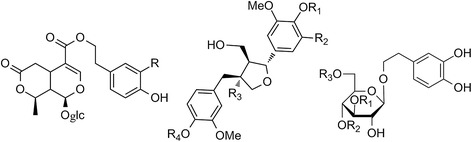
Phytochemical and pharmacological progress on the genus *Syringa*

Phytochemical and pharmacological progress on the genus *Syringa*

## Introduction

Plants belonging to the family Oleaceae, which consists of 27 genera and 400 species worldwide, have important applications in the daily life of people living in developing countries. Plants of many well-known genera, including *Forsythia*, *Syringa*, and *Osmanthus*, have been widely used for medicinal and industrial purposes. For instance, the stems and roots of *S. pinnatifolia* var. *alashanensis* is the major composition of atraditional formula ‘Ba wei chenxiang’ powder that is used for treatment of asthma, cardiopalmus, and angina [1].

Most *Syringa* plants are deciduous shrubs and arbors and include more than 40 species distributed around Europe and Asia [2]. At present, 22 species are found in China, of which 18 are endemic species that are mainly distributed in the southwestern part of Sichuan, Yunnan, Tibet, and other Northwestern regions. Many *Syringa* species, such as *S. chinensis*, *S. meyeri*, and *S. pekinensis*, are used for making ornaments. Flowers of *S. oblata* and *S. reticulata* var. *mandshurica* are an ideal source of aroma oils or nectar. Some *Syringa* plants are also used for construction purposes or for manufacturing furniture [1].

Previous phytochemical studies on *Syringa* species have revealed the presence of more than 140 secondary metabolites, including iridoids, lignans, phenylethanoids, their glycosides, minor organic acids, and essential oils [3,4]. Modern pharmacological studies have shown the bioactivities of these metabolites, such as antitumor, antihypertensive, anti-oxidant, anti-inflammatory activities, and so on [5]. However, a systematic review of these studies has not been performed to date. This review summarizes the phytochemical and pharmacological progress on *Syringa* to date by focusing on its chemical classification, structural features, and biological and pharmacological applications to provide information for further research on this genus.

### Chemical constituents

**Figure 1:**
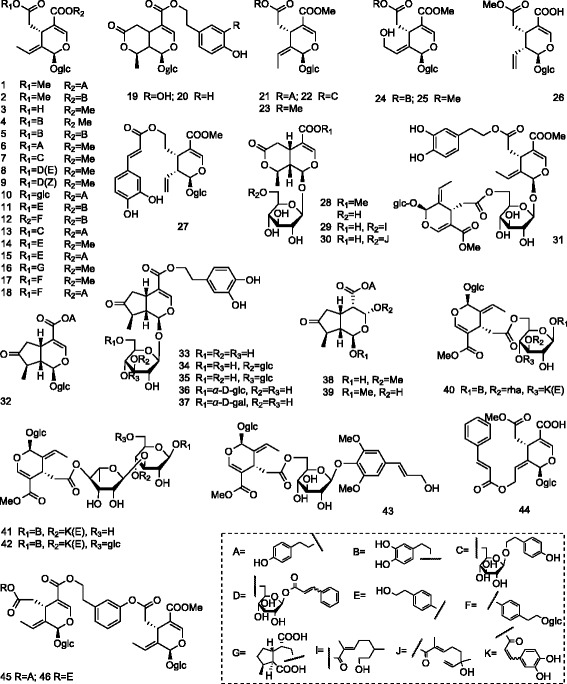
**The structures of iridoids from the genus** ***Syringa*** **.**

**Figure 2:**
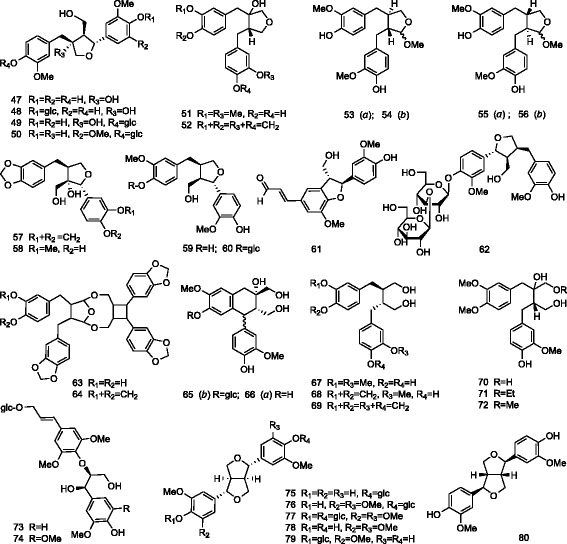
**The structures of lignans from the genus** ***Syringa*** **.**

**Figure 3:**
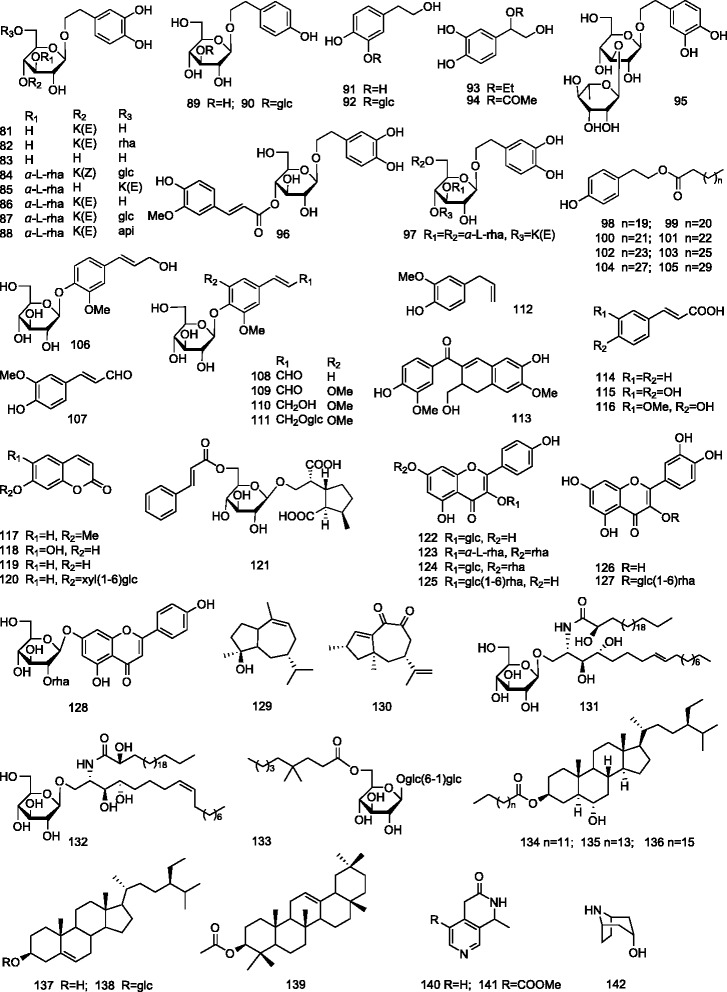
**The structures of other type of compounds from the genus** ***Syringa*** **.**

Previous studies have reported that extracts of *Syringa* plants contain iridoids (**1**–**46**), lignans (**47**–**80**), phenylpropanoids (**81**–**105**), phenylethanoids (**106**–**121**), and other compounds (**122**–**142**). The structures of these compounds are shown in Figures 1, 2, and 3 and related information are listed in Tables 1, 2, and 3.

**Table 1:** **Iridoids from the genus** ***Syringa***

**No**	**Compound**	**Part of plants**	**Source**	**Reference**
**1**	Isoligustroside	leaves	*S. vulgaris*	[11]
**2**	Isooleuropein	leaves	*S. vulgaris*	[11]
**3**	Oleoside 11-methyl ester	flowers, leaves and floral buds	*S. pubescens*	[3,5]
			*S. patula*	
**4**	Oleuropein	flowers, leaves, barks and floral buds	*S. pubescens*	[3,5,8,10,12-14]
			*S. reticulata*, *S. dilatata*, *S. velutina*, *S. afghanica*, *S. oblata* var. *alba*, *S. patula*	
**5**	Neooleuropein	leaves	*S. vulgaris*	[15]
**6**	8(*E*)-Ligstroside	flowers, leaves and barks	*S. pubescens*, *S. reticulata*, *S. dilatata*, *S. afghanica*	[3,8,10,13]
**7**	8(*E*)-Nüzhenide	leaves	*S. reticulata*	[16]
**8**	Safghanoside A	leaves	*S. afghanica*	[13]
**9**	Safghanoside B	leaves	*S. afghanica*	[13]
**10**	Safghanoside C	leaves	*S. afghanica*	[13]
**11**	Safghanoside D	leaves	*S. afghanica*	[13]
**12**	Safghanoside E	leaves	*S. afghanica*	[13]
**13**	Safghanoside F	leaves	*S. afghanica*	[13]
**14**	Formoside	leaves	*S. afghanica*	[13]
**15**	Fraxiformoside	leaves	*S. afghanica*	[13]
**16**	2''-*epi*-frameroside	leaves	*S. afghanica*	[13]
**17**	1'''-*O*-*β*-D-glucosylformoside	leaves	*S. afghanica*	[13]
**18**	1'''-*O*-*β*-D-glucosylfraxiformoside	leaves	*S. afghanica*	[13]
**19**	Lilacoside	barks and leaves	*S. vulgaris*	[17,18]
**20**	Fliederoside	barks and leaves	*S. vulgaris*	[17,18]
**21**	8(*Z*)-Ligstroside	leaves	*S. reticulata*	[16]
**22**	8(*Z*)-Nüzhenide	leaves	*S. reticulata*	[16]
**23**	Oleoside dimethyl ester	leaves	*S. afghanica*	[13]
**24**	10-Hydroxyoleuropein	flowers and leaves	*S. pubescens*	[3]
**25**	10-Hydroxyoleoside dimehyl ester	flowers and leaves	*S. pubescens*	[3]
**26**	Secologanoside 7-methyl ester	leaves	*S. reticulata*	[19]
**27**	Grandifloroside 11-methyl ester	flowers and leaves	*S. pubescens*	[3]
**28**	8-Epikingiside	barks	*S. vulgaris*	[20]
**29**	Syrveoside A	leaves	*S. velutina*	[21]
**30**	Syrveoside B	leaves	*S. velutina*	[21]
**31**	Jaspolyoside	barks	*S. reticulata*	[10]
**32**	Syringopicroside	leaves	*S. dilatata*, *S. vulgaris*, *S. oblata*, *S. reticulata*	[8,16,19,22,23]
**33**	Syringopicroside B	leaves	*S. vulgaris*	[9]
**34**	3′-*O*-*β*-D-glucopyranosylsyring-opicroside	leaves	*S. reticulata*	[16]
**35**	4′-*O*-*β*-D-glucopyranosylsyring-opicroside	leaves	*S .reticulata*	[16]
**36**	6′-*O*-*α*-D-glucopyranosylsyring-opicroside	leaves	*S. reticulata*	[16]
**37**	6′-*O*-*α*-D-galactopyranosylsyring-opicroside	leaves	*S. reticulata*	[19]
**38**	Syringopicrogenin C	seeds	*S. oblata*	[24]
**39**	Syringopicrogenin A	seeds and crust	*S. oblata*	[24,25]
**40**	Isooleoacteoside	leaves	*S. vulgaris*	[9]
**41**	Oleoacteoside	leaves	*S. reticulata*	[9,26]
**42**	Oleoechinacoside	leaves	*S. reticulata*	[9,26]
**43**	Reticuloside	barks	*S. reticulata*	[10]
**44**	Jasminoside	whole plant	*S. komarowii*	[27]
**45**	Safghanoside H	leaves	*S. afghanica*	[13]
**46**	Safghanoside G	leaves	*S. afghanica*	[13]

**Table 2:** **Lignans from the genus** ***Syringa***

**No**	**Name**	**Part of plant**	**Source**	**Reference**
**47**	(−)-Olivil	whole plant	*S. komarowii*	[27]
**48**	Olivil 4-*O*-*β*-D-glucopyranoside	barks	*S. reticulata*, *S. patula*	[10]
**49**	Olivil 4''-*O*-*β*-D-glucopyranoside	barks	*S. reticulata*	[27]
**50**	Armandiside	barks	*S. reticulata*	[10]
**51**	Syripinnalignan A	roots and stems	*S. pinnatifolia* var. *alashanensis*	[31]
**52**	Syripinnalignan B	roots and stems	*S. pinnatifolia* var. *alashanensis*	[31]
**53**	(8*R*, 8′*R*, 9*S*)-4-4′-dihydroxy-3, 3′, 9-trimethoxy-9-9′-epoxylignan	roots and stems	*S. pinnatifolia* var. *alashanensis*	[30]
**54**	(8*R*, 8′*R*, 9*R*)-4-4′-dihydroxy-3, 3′, 9-trimethoxy-9-9′-epoxylignan	roots and stems	*S. pinnatifolia* var. *alashanensis*	[30]
**55**	(8*S*, 8′*S*, 9*R*)-4-4′-dihydroxy-3, 3′, 9-trimethoxy-9-9′-epoxylignan	roots and stems	*S. pinnatifolia* var. *alashanensis*	[30]
**56**	(8*S*, 8′*S*, 9*S*)-4-4′-dihydroxy-3, 3′, 9-trimethoxy-9-9′-epoxylignan	roots and stems	*S. pinnatifolia* var. *alashanensis*	[30]
**57**	Mandshuricol A	leaves	*S. reticulata* var. *mandshurica*	[32]
**58**	Mandshuricol B	leaves	*S. reticulata* var. *mandshurica*	[32]
**59**	(+)-Lariciresinol	seeds crust	*S. oblata*	[25]
**60**	(+)-Lariciresinol 4-*O*-*β*-D-glucopyranoside	barks	*S. vulgaris*	[29]
**61**	Balanophonin	roots and stems	*S. pinnatifolia* var. *alashanensis*	[30]
**62**	(+)-Lariciresinol 4′-*O*-*β*-D-glucopyran -osyl-(1→3)-*β*-D-glucopyranoside	leaves	*S. reticulata*	[19]
**63**	Syripinnalignin A	roots and stems	*S. pinnatifolia* var. *alashanensis*	[33]
**64**	Syripinnalignin B	roots and stems	*S. pinnatifolia* var. *alashanensis*	[33]
**65**	Cycloolivil 6-*O*-*β*-D-glucoside	barks	*S. reticulata*	[10]
**66**	(+)-Cycloolivil	whole plant	*S. komarowii*	[27]
**67**	(−)-Secoisolariciresinol	stems	*S. pinnatifolia* var. *alashanensis*	[30,31]
**68**	PiperphilippininVI	roots and stems	*S. pinnatifolia*	[30]
**69**	Dihydrocubebin	roots and stems	*S. pinnatifolia* var. *alashanensis*	[30]
**70**	Syripinnalignan C	roots and stems	*S. pinnatifolia* var. *alashanensis*	[34]
**71**	Syripinnalignan D	roots and stems	*S. pinnatifolia* var. *alashanensis*	[34]
**72**	Syripinnalignan E	roots and stems	*S. pinnatifolia* var. *alashanensis*	[34]
**73**	(7*S*, 8*R*)-Guaiacylglycerol-8-*O*-4′-sinapyl ether 9′-*O*-*β*-D-glucopyranoside	leaves	*S. velutina*	[28]
**74**	(7*S*, 8*R*)-Syringylglycerol-8-*O*-4′-sinapyl ether 9′-*O*-*β*-D-glucopyranoside	leaves	*S. velutina*	[28]
**75**	Pinoresinol-4-*O*-*β*-monoglycoside	barks	*S. reticulata*	[10]
**76**	Syringaresinol-4-*O*-*bis*-*β*-D-monoglucoside	barks	*S. reticulata*	[10]
**77**	Syringaresinol-4, 4''-*O*-*bis*-*β*-D-glucoside	barks	*S. reticulata*	[10]
**78**	Syringaresinol	floral buds, flowers and leaves	*S. patula*, *S. pubescens*	[3,5]
**79**	(+)-Medioresinol-4-*O*-glucoside	floral buds	*S. patula*	[5]
**80**	(−)-Pinoresinol	roots and stems	*S. pinnatifolia* var. *alashanensis*	[30]

**Table 3:** **Other type of compounds from the genus** ***Syringa***

**No**	**Name**	**Part of plant**	**Source**	**Reference**
**81**	Isosyringalide	leaves	*S. reticulata*	[41]
**82**	Forsythiaside	barks	*S. vulgaris*	[29]
**83**	2-(3, 4-dihydroxy)-phenylethyl-*β*-D-glucopyranoside	barks	*S. reticulata*	[10,12]
**84**	*cis*-Echinacoside	leaves	*S. reticulata*	[35]
**85**	Isoverbascoside	leaves	*S. pubescens*	[3]
**86**	Verbascoside	leaves	*S. pubescens*, *S. oblata* var. *alba*, *S. vulgaris*	[3,9,29,14,36]
**87**	Echinacoside	barks, leaves and flowers	*S. pubescens*, *S. reticulata*	[3,29,42]
			*S. vulgaris*	
**88**	Forsythoside B	leaves	*S. reticulata* var. *mandshurica*	[35]
**89**	Salidroside	barks	*S. reticulata*	[10]
**90**	3′-*O*-*β*-D-glucopyranosysalidroside	leaves	*S. reticulata* var. *mandshurica*	[35]
**91**	2-(3, 4-dihydroxyphenyl) ethanol	leaves	*S. pubescens*	[3]
**92**	Osmanthuside F	leaves	*S. reticulata*	[35]
**93**	(*S*)-(+)-2-(3, 4-dihydroxyphenyl)-2-ethoxylethanol	leaves	*S. reticulata* var. *mandshurica*	[43]
**94**	(*S*)-(+)-2-(3, 4-dihydroxyphenyl)-2-acetoxyethanol	leaves	*S. reticulata* var. *mandshurica*	[43]
**95**	Decaffeoylacteoside	leaves	*S. reticulata*	[35]
**96**	Syringalide B	leaves	*S. reticulata*	[41]
**97**	Poliumoside	leaves	*S. afghanica*	[13]
**98**	2-(4-hydroxypenyl)-ethyl behenate	whole plant	*S. komarowii*	[27]
**99**	2-(4-hydroxypenyl)-ethyl tricosanoate	whole plant	*S. komarowii*	[27]
**100**	2-(4-hydroxypenyl)-ethyl lignocerate	whole plant	*S. komarowii*	[27]
**101**	2-(4-hydroxyhenyl)-ethyl pentacosanoate	whole plant	*S. komarowii*	[27]
**102**	2-(4-hydroxypenyl)-ethyl hexacosanoate	whole plant	*S. komarowii*	[27]
**103**	Bongardol	whole plant	*S. komarowii*	[27]
**104**	2-(4-hydroxypenyl)-ethyl 1-dodecyloctadecanoate	whole plant	*S. komarowii*	[27]
**105**	2-(4-hydroxypenyl)-ethyl dotriacontanoate	whole plant	*S. komarowii*	[27]
**106**	Coniferin	barks	*S. vulgaris*	[29]
**107**	Coniferylaldehydel	roots and stems	*S. pinnatifolia* var. *alashanensis*	[44]
**108**	Coniferyaldehyde glucoside	barks	*S. reticulata*	[10]
**109**	Sinapaldehyde glucoside	barks	*S. reticulata*	[10]
**110**	Syringin	barks	*S. vulgaris*, *S. reticulata*	[10,45,46]
**111**	Isosyringinoside	barks	*S. reticulata*	[10]
**112**	Eugenol	foral buds	*S. patula*	[5]
**113**	Larixnaphthanoe	roots and stems	*S. pinnatifolia* var. *alashanensis*	[30]
**114**	Cinnamic acid	leaves, roots and stems	*S. afghanica*, *S. pinnatifolia* var. *alashanensi*, *S. reticulata*	[44,47]
**115**	Caffeic acid	roots and stems	*S. pinnatifolia* var. *alashanensis*	[44]
**116**	Ferulic acid	roots and stems	*S. pinnatifolia* var. *alashanensis*	[44]
**117**	7-Methoxycoumarin	roots and stems	*S. pinnatifolia* var. *alashanensis*	[44]
**118**	Esculetine	roots and stems	*S. pinnatifolia* var. *alashanensis*	[44]
**119**	Umbelliferone	roots and stems	*S. pinnatifolia* var. *alashanensis*	[44]
**120**	*O*-[*β*-D-xylopyanosy (1–6) *β*-D-glucopyranosyl]-7-hydroxycoumarin	roots and stems	*S. pinnatifolia* var. *alashanensis*	[44]
**121**	Syringfghanoside	leaves	*S. afghanica*	[13]
**122**	Astragalin	bark	*S. vulgaris*	[48]
**123**	Kaempferol-3, 7-*α*-L-dirhamnoside	flowers and leaves	*S. pubescens*	[3]
**124**	Kaempferol-3-*β*-D-glucoside-7-*α*-L-dirhamnoside	flowers and leaves	*S. pubescens*	[3]
**125**	Kaempferol-3-*O*-rutinoside	flowers	*S. vulgaris*	[49]
**126**	Luteolin	leaves	*S. afghanica*	[13]
**127**	Rutin	leaves	*S. vulgaris*	[49,50]
**128**	Rhoifolin	leaves	*S. afghanica*	[13]
**129**	Guai-9-en-4*β*-ol	roots and stems	*S. pinnatifolia* var. *alashanensis*	[37]
**130**	14, 15-dinorguai-1, 11-dien-9, 10-dione	roots and stems	*S. pinnatifolia* var. *alashanensis*	[37]
**131**	Momorcerebroside I	whole plant	*S. komarowii*	[27]
**132**	Phytolacca cerebroside	whole plant	*S. komarowii*	[27]
**133**	Pubescenside A	flowers and leaves	*S. pubescens*	[51]
**134**	Stigmastane-3*β*, 6*α*-diol 3-*O*-tetradecanoate	whole plant	*S. komarowii*	[27]
**135**	Stigmastane-3*β*, 6*α*-diol 3-*O*-palmitate	whole plant	*S. komarowii*	[27]
**136**	Stigmastane-3*β*, 6*α*-diol 3-*O*-stearate	whole plant	*S. komarowii*	[27]
**137**	*β*-sitosterol	foral buds and whole plant	*S. patula*, *S. komarowii*	[5,27]
**138**	Daucosterol	whole plant	*S. komarowii*	[27]
**139**	*β*-Amyrin acetate	foral buds	*S. patula*	[5]
**140**	Jasminidin	leaves	*S. vulgaris*	[52]
**141**	Jasminin	leaves	*S. vulgaris*	[52]
**142**	Nortropin	foral buds	*S. patula*	[5]

### Iridoids

Iridoids are one of the most important natural compounds that are widely distributed in various plant families such as Plantaginaceae, Rubiaceae, and Scrophulariaceae [6]. Iridoids are extensively present in almost all *Syringa* species and have antitumor, antihypertensive, anti-inflammatory, anti-oxidant, and antifungal activities. In addition, iridoids play an important role in defense mechanism of ants [7]. Among all the iridoids reported in this genus, secoiridoids are the most abundant and have been shown to have antitumor activity. To date, 46 iridoids (**1**–**46**) have been described, including secoiridoids (**1**–**30** and **40**–**44**), eight typical iridoids (**32**–**39**), and three minor dimers (**31**, **45**, and **46**). Most iridoids exist as glycosides and are mainly produced by the glycosylation of glucose and galactose. *Syringa* iridoids are generally substituted by various acid fragments and phenolic moieties such as 1-*O*-cinnamoyl-*β*-d-glucopyranosyl, *p*-hydroxphenethyl, 3, 4-dihydroxy-phenethyl, and caffeic acid, which contribute to their low polarity. *Syringa* iridoids have antitumor (**33** and **40**) [8,9], antihypertensive (**4**), and anti-oxidant (**4** and **31**) activities [10].

### Lignans

Lignans are another major compounds in this genus, particularly in *S. komarowii* [27], *S. pubescens* [3], *S. reticulata* [10], *S. velutina* [28], *S. patula* [5], *S. vulgaris* [29], *S. pinnatifolia* var. *alashanensis* [30,31], and *S. reticulata* var. *mandshurica* [32]. *Syringa* species have 34 lignans and their glycosides (**47**–**80**), including monoepoxylignans (**47**–**60**, **62**) and their dimers (**63** and **64**), neolignans (**61**, **73**–**74**), cyclolignans (**65** and **66**), simple lignans (**67**–**72**), and bisepoxylignans (**75**–**80**). Lignans also exhibit many bioactivities. For example, compound **50** has anti-oxidant activity [10]; compounds **57** and **58** have antifungal activities [32]; and compound **75** has significant cytotoxic, antihypertensive, anti-inflammatory, and anti-oxidant activities [5].

### Other compounds

Phenylethanoids (**81**–**105**), phenylpropanoids and their analogues (**106**–**121**), flavonoids (**122**–**128**), sesquiterpenes (**129** and **130**), and other minor compounds have been described in *Syringa* plants. Of these, phenylethanoids are predominant, particularly in *S. reticulata* [10,12,35], *S. vulgaris* [29], *S. pubescens* [3], *S. oblata* var. *alba* [36], *S. reticulata* var. *mandshurica* [35], *S. afghanica* [13], and *S. komarowii* [27]. Sesquiterpenes (**129** and **130**) are present in the stems of *S. pinnatifolia* var. *alashanensis* [37]. These miscellaneous compounds have cytotoxic, anti-inflammatory, antihypertensive, anti-oxidant, and antifungal properties.

Besides the abovementioned compounds, *Syringa* plants contain essential oils that form the most important constituents not only because of their economic utility but also because of their potential medicinal value as antimicrobial, antipyretic, and antiviral agents. Multiple analytical techniques such as headspace solid-phase microextraction, gas chromatography–mass spectrometry (GC–MS), GC–MS coupled with heuristic evolving latent projections, moving subwindow searching, nuclear magnetic resonance spectroscopy, and X-ray single-crystal diffraction analysis have been used to identify essential oils from fresh flowers of *S. oblata* var. *alba*. For instance, 39 volatile oil constituents were identified, including four characteristic isomers of lilac alcohols (lilac alcohols A–D) and lilac aldehydes A–D [38]. Ninety-five components, including 15 terpenes, 14 oxygenated terpenes, 10 aromatic compounds, and 13 *n*-alkanes were quantitatively analyzed from *S. oblata* buds [39]. Forty-nine components were described from essential oil of *S. pubescens* flowers, most of which are monoterpenes and sesquiterpenes [40]. Thirty-four volatile oil components, accounting for around 64.7% (zerumbone) of the toil oil, were identified from roots and barks of *S. pinnatifolia* var. *alashanensis* [4]. These data imply that *Syringa* plants could be considerably different from each other in terms of their essential oil components.

### Pharmacological activities

Various crude extracts and isolated compounds from *Syringa* plants have shown significant antitumor, antihypertensive, anti-inflammatory, anti-oxidant, and antifungal activities.

### Antitumor activity

Cytotoxic activities of crude extracts and chemicals obtained from *Syringa* plants have been extensively evaluated against various tumor cell lines. Aqueous extracts from the flowers and leaves of *S. pubescens* inhibited the growth of L2215 (hepatitis B virus) cells, with a 50% inhibitory concentration (IC_50_) value of 78 *μ*g/mL [51]. Hydrolysis of isoligustroside (**1**) and isooleuropein (**2**) were assayed using a disease-oriented panel of 39 human cancer cell lines. The results showed that the hydrolysis product of compound **2** had moderate cytotoxic activity against lung cancer cell lines DMS273 [log GI_50_ = 5.19 (6.4 *μ*M)] and DMS114 [log GI_50_ = 5.06 (8.7 *μ*M)]. Preliminary analysis of structure–activity relationship suggested that C-5′-OH plays an important role in this cytotoxic activity [11]. Isooleoacteoside (**40**) showed weak cytotoxicity against LOX-IMVI melanoma cell line, with GI_50_ value of 16 *μ*M, and syringopicroside B (**33**) showed weak cytotoxic activity against NCI-H522 lung cancer cell line, with GI_50_ value of 13 *μ*M [9]. MTT assay used to assess the cytotoxicities of syringaresinol (**78**) and oleoside 11-methyl ester (**3**) showed that compound **78** had a strong dose-dependent effect on HepG2 cell line, with an IC_50_ value of 94.6 *μ*M, and compound **3** has a dose–response curve of low slope, with a high IC_50_ value of 186.5 *μ*M, compared with positive controls dexamethasone (IC_50_ 14.2 *μ*M) and paclitaxel (IC_50_ 700 nM). However, compound **78** was cytotoxic even at the lowest concentration of 29.9 *μ*M. *β*-Amyrin acetate (**139**) showed weak cytotoxicity against A2780 human ovarian cancer and HepG2 cell lines [5]. Oleuropein (**4**) and 2-(3, 4-dihydroxy)-phenylethyl-*β*-d-glucopyranoside (**83**) showed evident cytotoxicities against P-388, L-1210, SNU-5, and HL-60 cell lines, with IC_50_ values varying from 8.5 to 139.8 *μ*M [12]. Verbascoside (**86**) showed moderate cytotoxic activity against SNB-75 (brain cancer) and SNB-78 cell lines, with GI_50_ values of 7.4 and 7.7 *μ*M, respectively [9]. A pharmacokinetic study showed that compound **86** interacted with the catalytic domain of PKC and acted as a competitive inhibitor of adenosine triphosphate (K_i_ = 22 *μ*M) and non-competitive inhibitor of phosphate acceptor (histone III). Because **83** is one part of **86** in its molecular structure, the cytotoxic effect could be attributed to 3, 4-dihydroxyphenylethoxy moiety, which may act as a competitive inhibitor to the catalytic domain of PKC. Therefore, **83** is a potentially essential skeleton of most cytotoxic phenylethanoid glycosides [12].

### Hypotensive activity

Syringin (**110**) and kaempferol-3-*O*-rutinoside (**125**) showed antihypertensive activity. Intravenous injection of 10 mg/kg of compound **86** significantly decreased systolic, diastolic, and mean arterial blood pressure in Pentothal-anesthetized rats. Moreover, the depressor effect of compound **86** was independent of muscarinic and histaminergic receptors because it did not block the effect of atropine (an antimuscarinic agent) and chlorpheniramine/cimetidine (antihistaminergic agents) [36]. *In vitro* studies showed that oleuropein (**4**) significantly lowered blood pressure. It is interesting to note that antihypertensive effect of compound **4** (33% at 30 mg/kg dose) on the blood pressure of anesthetized rats was similar to that of compound **86** (39.04% ± 2.38% at 10 mg/kg dose) [14,36], which is probably because of the similarity in their structures, with both possessing the same aromatic fragment having two hydroxy groups.

### Anti-inflammatory activity

Iridoid glycosides (IGs) exerted obvious anti-inflammatory effects on ulcerative colitis *in vivo* by inhibiting relative proinflammatory cytokines [53]. IGs significantly ameliorated macroscopic damages and histological changes, reduced the activity of myeloperoxidase, and strongly inhibited epithelial cell apoptosis. Moreover, IGs markedly decreased the levels of tumor necrosis factor-*α*, interleukin-8, cyclooxygenase-2, and transforming growth factor-*β*1 in colonic tissues in a dose-dependent manner. Moreover, effects of IGs (160 and 240 mg/kg) were superior to those of positive control salicylazosulfapyridine (150 mg/kg). Furthermore, IGs significantly blocked NF-κB signaling by inhibiting inflammatory bowel phosphorylation/degradation and inhibitor kappa B kinase *β* activity; downregulated protein and mRNA expressions of Fas/FasL, Bax, and caspase-3; and activated Bcl-2 in intestinal epithelial cells [53,54]. *β*-Amyrin acetate (**139**) and syringaresinol (**78**) at a dose of 20 *μ*g/mL evidently inhibited lipopolysaccharide-induced nitric oxide (NO) production, with inhibition rates of 49.97% and 33.21%, respectively [5].

### Liver-protective and cholagogic effects

Crude extract of *Syringa* species, interferon (IFN), and an injection of “Gan-Yan-Ling” were compared to evaluate their liver-protective effects on the survival rates of HepG2.215 cells and secretion of hepatitis B surface antigen (HBsAg) and HBeAg. The results indicated that all the three assayed drugs may suppress the secretion of HBsAg and HBeAg from HepG2.215 cells in a dose-dependent manner, with the effect of crude extract of *Syringa* being intermediate those of IFN and Gan-Yan-Ling. Therefore, extracts of *Syringa* plant could be used to develop effective and less toxic antihepatitis B medicines [55].

Aqueous extracts of *S. reticulata* var. *mandshurica* significantly decreased the levels of alanine transaminase and aspartate transaminase and the concentration of malondialdehyde in the serum but increased the activity of superoxide dismutase (SOD) in the liver. These extracts showed protective effects on acute liver injury induced by CCl_4_ in mice [56]. In addition, the essential oils of *Syringa* exerted protective effects on the liver and cholecyst [39].

### Antifungal activity

Phenylpropanoids such as verbascoside (**86**) and forsythiaside (**82**) exhibit significant antimicrobial activity [29]. Compounds **93** and **94** at 1- mM concentration inhibited the radial growth of *Phytophthora capsici* after 6 days of incubation, with inhibition rates 59.1% and 72.5%, respectively [43]. Two sesquiterpenes, guai-9-en-4*β*-ol (**129**) and 4, 15-dinorguai-1, 11-dien-9, 10-dione (**130**), have antibacterial and antifungal properties. Compound **129** was active against *Bacillus coagulans* [inhibition zone (IZ) = 15.34 mm] and *Aspergillus niger* (IZ = 13.20 mm) while compound **130** significantly inhibited *Escherichia coli* (IZ = 15.34 mm) and *Fusarium oxysporum* (IZ = 15.32 mm) [37].

Compound **3** showed effective antimicrobial activity against *Lactobacillus pentosus* (IZ = 1 mm), and compound **139** inhibited the growth of *Candida* species at concentrations of 30–250 *μ*g/mL [5].

### Antioxidant activity

A 70% EtOH extract of *S. reticulata* barks showed potent superoxide anion and DPPH free radical scavenging activities, with EC_50_ values of 5.88 and 38.10 *μ*g/mL, respectively [10].

Among the compounds isolated from the bark of *S. reticulata*, six (**4**, **31**, **50**, **77**, **83**, and **111**) showed significant superoxide anion scavenging activity, with EC_50_ values of 2.57, 4.97, 10.64, 15.98, 4.97, and 14.14 *μ*g/mL, respectively. Compound **4** also interacted with the stable free radical DPPH, with an IC_50_ value of 40.4 *μ*M [8,10]. These different anti-oxidant activities are closely related to their structural features. Presence of 2-(3, 4-dihydroxyphenyl)-ethoxy moiety might be important for a higher activity because the most potent compounds (EC_50_ = 2.57–4.97 *μ*M), including the two secoiridoid glycosides (**31** and **4**) and a phenylethanoid glycoside (**83**), possess the same structural features. Comparison of the structures of compounds **4** and **83** with those of 8(*Z*)-ligstroside (**21**) and salidroside (**89**) showed that presence of *ortho*-coupling hydroxyl group at C-2 might be responsible for their different activities. It has been previously reported that 1, 2-dihydroxybenzene moiety is crucial to its DPPH scavenging activity [10].

Syringaresinol (**78**) showed a strong scavenging activity against DPPH, with EC_50_ value as low as 12.5 *μ*g/mL, which might be responsible for its strong inhibition of NO production [5].

Eugenol (**112**) inhibited the catalytic activity of H_2_O_2_/Ca^2+^ human erythrocyte membrane lipid peroxidation at a concentration of 200 *μ*mol/L, with an inhibition rate of 62%, and completely suppressed the catalytic activity of dibenzoyl peroxide/Ca^2+^ human erythrocyte membrane lipid peroxidation at a concentration of 100 *μ*mol/L. Compound **112** exerted its effect in a non-competitive manner by reacting with Ca^2+^ and inhibiting the formation of hydroxyl radicals, thus, protecting the cell membrane lipid from oxidation [2].

### Inhibition of platelet aggregation

Aqueous extract of *S. aramaticum* significantly inhibited adenosine diphosphate (ADP) and collagen-induced platelet aggregation, with inhibition rates of 37.4% and 69.7%, respectively [57]. Mandshuricols A (**57**) and B (**58**) showed antagonistic activities on platelet-activating factor (PAF) in [3H]PAF receptor binding assay, with IC_50_ values of 4.8 × 10^−5^ and 3.5 × 10^−5^ M, respectively [32].

### Others

Essential oils from the stems and roots of *S. pinnatifolia* var. *alashanensis* (SPEO) reduced the deviation of ST segment; decreased the levels of lactate dehydrogenase, creatine kinase, and troponin T; and increased the activity of SOD. These protective effects were further confirmed by histopathological examination [58]. Treatment with both 8 and 32 mg/kg SPEO prolonged the survival of mice under hypoxia conditions, showing a remarkable protective effect against H_2_O_2_-induced death in cultured rat myocytes. Moreover, 5, 2.5 and 1.25 *μ*g/mL doses of SPEO inhibited ADP-induced rat platelet aggregation by 47.4%, 37.0%, and 32.9%, respectively [58], implying that SPEO exerted protective effects against myocardial ischemia.

Oral and intraperitoneal administration of 0.2–0.4 g of leaf extract of *S. vulgaris* in cats or rabbits exerted an antipyretic effect that was equal to the effect of 0.1–0.3 g of aminopyrine administered orally or intraperitoneally. However, leaf extracts of *S. vulgaris* are considerably more toxic than aminopyrine, with their toxic dosages being 0.4 and 1.2 g/kg, respectively [59]. *In vitro* evaluation of leaf extract of *S. aramaticum* showed its antiviral activity against herpes simplex virus at concentrations 1.25%–2.5%. The protective effect was more obvious when controlling the amount of virus attacks at 9.2–92 tissue culture infective dose (TCID50), suggesting that *S. aramaticum* effectively killed the virus without any harmful side effects [60-62].

Studies have reported that leaf extracts of *S. aramaticum* could be used for treating hemorrhoids [63]. Eugenol (**112**) inhibited the metabolism of arachidonic acid. Extracts of *S. reticulata* var. *mandshurica* have been used for treating bronchitis, and one of its constituents 2-(3, 4-dihydroxyphenyl) ethanol (**91**) significantly inhibited the production of phlegm [2].

## Review and conclusions

This review describes phytochemical and pharmacological progress on the genus *Syringa* in the recent 20 years and discusses the future research prospects.

*Syringa* plants are used not only as traditional medicines to treat rheumatoid arthritis, asthma, cardiopalmus, and angina pectoris by natives in China but also for making ornaments, volatile oils, food additives, and bactericides worldwide, particularly in developing countries. Previous phytochemical studies on crude extracts from various species of this genus have identified iridoids, lignans, phenylpropanoids, and phenylethanoids having antitumor, antihypertensive, anti-oxidant, and anti-inflammatory activities. Iridoids, lignans, and phenylethanoids are the most predominant compounds in *Syringa* plants that probably contribute independently or synergistically to their main biological activities.

To the best of our knowledge, 46 iridoid representatives have been reported in *Syringa* plants, with high concentrations present in the leaves of *S. vulgaris*, *S. pubescens*, *S. afghanica*, *S. reticulata*, and *S. velutina* and barks of *S. vulgaris* and *S. reticulata* and low concentrations present in the flowers (*S. pubescens*), seeds, and seeds crust (*S. oblata*). This difference may be associated with their ecological roles, because iridoids are produced mainly to fight predators and/or microbes. Moreover, high concentrations of lignans in the stems and roots can be attributed to the rigidity of these plants. This may be the reason for the absence of iridoids in *S. pinnatifolia* var. *alashanensis* because materials used for chemical investigation included peeled stems and roots. Anti-inflammatory effects of extracts from these plants are mainly responsible for their applications in traditional medicine. However, only preliminary work has been performed on most isolated compounds, such as *in vitro* cytotoxicity screening (**1**, **2**, **78**, and **139**). Limited studies have been performed on the *in vivo* effects of these compounds; thus, providing opportunities for further detailed research. It is particularly worthy to mention that China has an abundant resource of *Syringa*, with many endemic species. For instance, *S. pinnatifolia* var. *alashanensis* is a well-known Mongolian medicine traditionally used for myocardial ischemia in clinical practice. However, no substantial evidence is available on its bioactive ingredients and mechanisms of action underlying this effect. Therefore, it deserves further phytochemical and pharmacological studies.
